# Myoepithelioma-like tumor of the vulvar region with estrogen receptor negativity and *GSTT1::IGLC7* fusion: a case report

**DOI:** 10.3389/fonc.2026.1743994

**Published:** 2026-04-13

**Authors:** Bei Wang, Tingting Song, Ajiguli Yilijiang, Jiangbo Wang, Shuxia Bai, Siqi Wang, Shaobo Liu, Wenjia Sun

**Affiliations:** 1 Department of Pathology, the People's Hospital of Bortala Mongolian Autonomous Prefecture, Bole, China; 2 Department of Pathology, Hubei Cancer Hospital, Tongji Medical College, Huazhong University of Science and Technology, Wuhan, China

**Keywords:** ER, GSTT1::IGLC7, MELTVR, NGS, SMARCB1, vulva

## Abstract

Myoepithelioma-like tumor of the vulvar region (MELTVR) is a rare mesenchymal neoplasm that exhibits morphological features resembling those of soft tissue myoepitheliomas, yet displays distinct immunophenotypic and molecular genetic characteristics. All MELTVR cases reported in the existing literature have been estrogen receptor (ER)-positive. In contrast, this study presents the first documented case of ER-negative MELTVR, thereby broadening the known immunohistochemical phenotypic spectrum of this neoplasm. Next-generation sequencing (NGS) analysis revealed copy number loss of *SMARCB1* and a novel *GSTT1::IGLC7* fusion in the tumor, while no other gene rearrangements, including those involving *EWSR1*, *NR4A3*, or *FUS*, were detected. This finding not only expands the molecular genetic spectrum of MELTVR but also provides valuable insight for future investigations into its pathogenesis, biological behavior, and refinement of clinical diagnostic and therapeutic strategies for this rare tumor.

## Introduction

Myoepithelioma-like tumor of the vulvar region (MELTVR) is a rare mesenchymal tumor that arises in the superficial subcutaneous tissue of the female vulva. The term was first coined by Yoshida et al. (1) in 2015 to describe a group of vulvar tumors that, while morphologically similar to soft tissue myoepitheliomas, exhibit incomplete concordance in their immunophenotypic and molecular genetic profiles with those of typical myoepitheliomas.

To date, only a small number of cases have been documented in both domestic and international literature, resulting in limited clinical and pathological understanding of its clinicopathological features, molecular expression profile, diagnostic and differential diagnostic criteria, and therapeutic management. The present study reports a case of estrogen receptor (ER)-negative MELTVR that underwent molecular analysis using next-generation sequencing (NGS). Together with a review of relevant literature, this report aims to enhance clinicians’ and pathologists’ understanding of this rare neoplasm.

## Case report

A 72-year-old female patient was admitted to the hospital on July 7, 2025, due to a “vulvar mass with progressive enlargement over six months.” The patient had no prior history of malignancy. Physical examination revealed a palpable mass approximately 5 × 6 cm in size, located in the upper segment of the left labia majora. The mass had relatively well-defined margins, poor mobility, a soft texture, and mild tenderness. Following completion of preoperative evaluations, the tumor was completely excised on July 10, 2025.

**Figure 1:**
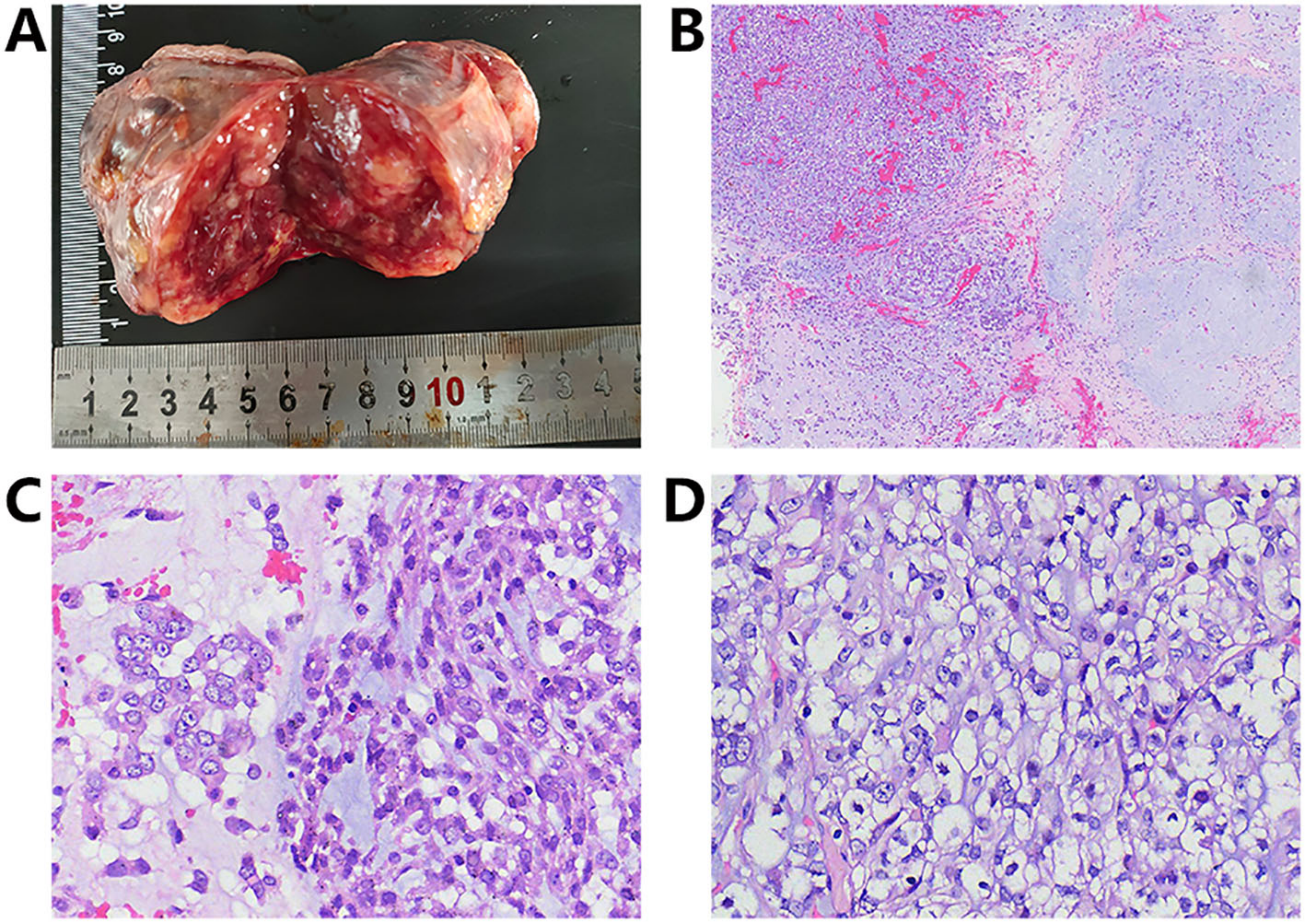
**(A)** The mass presented as a well-circumscribed oval lesion and the cut surface appeared grayish-red to grayish-white with a translucent, myxoid appearance; **(B)** The stroma demonstrated extensive myxoid alteration, comprising both hypercellular (left) and hypocellular (right) regions; **(C)** The tumor cells exhibited two distinct morphological patterns, namely epithelioid (left) and spindle (right) cell types; **(D)** Focal areas displayed clear cytoplasm.

Gross examination revealed a subcutaneous oval mass measuring 9.5 × 5.0 × 4.5 cm, with relatively well-defined boundaries and no ulceration of the overlying skin. The cut surface appeared grayish-red to grayish-white with a translucent, myxoid appearance, and a moderately firm consistency (**Figure 1A**).

Histologically, at low magnification, the tumor was well-circumscribed and partially encapsulated by a fibrous pseudocapsule. The stroma was richly vascularized and exhibited extensive myxoid change (accounting for approximately 95% of the tumor volume), containing alternating hypocellular and hypercellular areas (**Figure 1B**). At high magnification, two distinct cellular morphologies were observed (**Figure 1C**). Approximately 95% of the tumor cells were epithelioid with abundant, predominantly amphophilic cytoplasm, while focal regions displayed clear cytoplasm (**Figure 1D**). These epithelioid cells had vesicular nuclei with prominent nucleoli, mild to moderate nuclear pleomorphism, and occasional eccentric nuclei resembling plasma cells. The remaining 5% of cells, confined to focal regions, were spindle-shaped with inconspicuous nucleoli. The tumor cells were distributed singly or arranged in cords, clusters, loose networks, or solid sheets. Mitotic figures were observed at 10 per 10 high-power fields (HPF), and necrosis was present.

**Figure 2:**
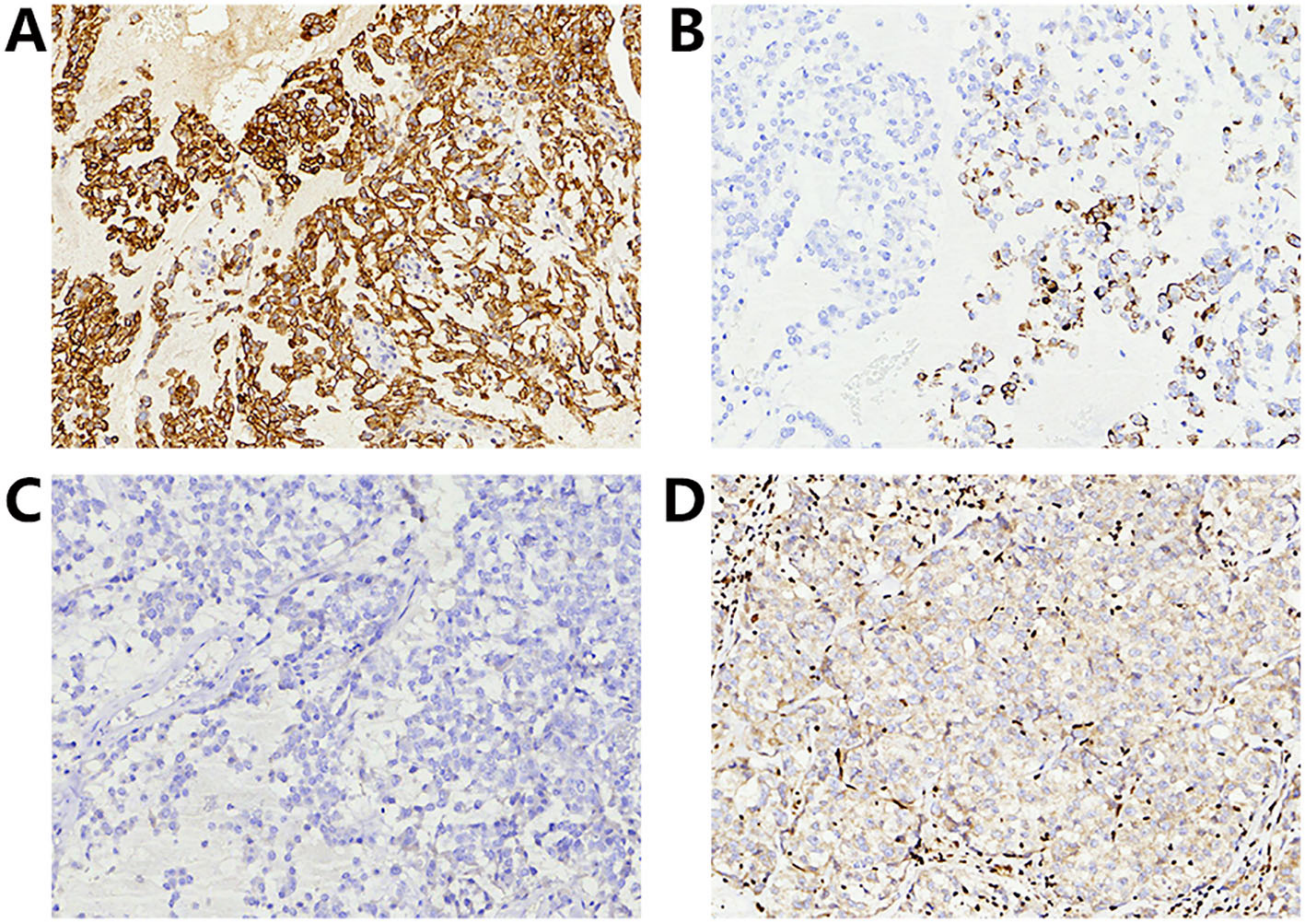
Immunohistochemical findings: **(A)** EMA; **(B)** CK; **(C)** ER; **(D)** INI1/*SMARCB1* (All tumor cells showed no INI1/*SMARCB1* expression, while endothelial cells expressed INI1/*SMARCB1*).

Immunohistochemically, the tumor cells demonstrated diffuse positivity for epithelial membrane antigen (EMA) (**Figure 2A**) and Vimentin, and partial positivity for cytokeratin (CK) (**Figure 2B**), S-100, smooth muscle actin (SMA) and Calponin. The tumor was negative for ER (**Figure 2C**), progesterone receptor (PR), CEA, cytokeratin 7 (CK7), CK5/6, P63, SOX10, CD34 and Desmin. P53 expression was heterogeneous, and Rb expression was retained. Complete loss of INI1/*SMARCB1* expression was observed in all tumor cells (**Figure 2D**). The Ki-67 labeling index was approximately 40%.

Comprehensive 425-gene NGS analysis revealed copy number deletion in the *SMARCB1* gene, a tumor mutational burden (TMB) of 0 mutation/Mb (TMB-low, TMB-L), and a *GSTT1::IGLC7* gene fusion. No additional genetic alterations were detected.

The pathological diagnosis was myoepithelioma-like tumor of the vulva (left labia majora). During a four-month follow-up period after surgical excision, no evidence of local recurrence or metastatic disease was observed.

## Discussion

MELTVR predominantly affects adult females, with a reported age range of 24 to 70 years (2). Clinically, it usually presents as a painless, slow-growing vulvar mass (3). The tumor primarily arises in the subcutaneous tissue of the perineal region, including the labia majora, mons pubis, inguinal area, and perianal region (4).

Grossly, MELTVR usually presents as a well-circumscribed nodule (1). Microscopically, the tumor is often partially enclosed by a fibrous pseudocapsule and divided into lobules by incomplete fibrous septa. In some cases, minimal extracapsular invasion or extensive infiltration into adjacent adipose tissue is observed (5). The tumor cells are epithelioid or spindle-shaped, with eosinophilic or amphophilic cytoplasm. Nuclei are uniform in size with mild-to-moderate atypia. Mitotic rate varies among cases and may be high, ranging up to 23 mitotic figures per 10 HPF (6). The tumor stroma is richly vascularized, comprising myxoid and non-myxoid components, with the myxoid component accounting for 5% to 95% of the total tumor volume (1). MELTVR exhibits morphological variability. In one case, the tumor showed a diffuse myxoid background with multinucleated giant cell–like tumor cells (7). Another case was histologically classified as a non-myxoid spindle-cell tumor resembling solitary fibrous tumor (8).

According to the literature, MELTVR exhibits a relatively distinct immunophenotype. It typically expresses Vimentin, EMA, ER, and PR, whereas SMA and Calponin may be expressed in a subset of cases. In contrast, S-100, P63, SOX10, CD34, glial fibrillary acidic protein (GFAP), Desmin, myogenin, CK, and CK7 are usually negative. Notably, loss of INI1/*SMARCB1* expression is a consistent finding, and the Ki-67 index ranges from 1% to 35% (3).

The immunohistochemical results of the present case are largely consistent with the literature, except for one critical discrepancy: complete loss of ER and PR expression, which has not been previously reported in MELTVR. In all prior studies, MELTVR consistently demonstrated ER and PR positivity to varying degrees, most commonly diffuse or partial positivity. Only one case exhibited strong positivity restricted to an extremely focal area (<1%) (5). Negative ER and PR expression may lead to diagnostic challenges in MELTVR. To exclude technical error, we retested an alternative paraffin block and validated the reliability of ER and PR negativity using multi-tissue external controls including uterine myometrium. These findings indicate that ER/PR positivity is not an invariable immunophenotypic feature of MELTVR.

At the molecular level, this case is consistent with previously reported MELTVR cases by harboring *SMARCB1* deletion. A recent genomic study identified *PPP6R3::FHDC1* and *MYH9::MYH6* fusions in two of three cases (9). In contrast, the present case demonstrated a novel *GSTT1::IGLC7* fusion, suggesting that the molecular genetic heterogeneity of MELTVR may be greater than previously recognized. *GSTT1* (Glutathione S-Transferase Theta 1) is a key regulatory gene that governs detoxification metabolism and oxidative stress homeostasis (10). By contrast, *IGLC7* (Immunoglobulin Lambda Constant 7) belongs to the immunoglobulin gene family and encodes the B lymphocyte-specific lambda light chain constant region of antibodies (11). These two genes exhibit no known functional association under physiological conditions, and neither has previously been reported to participate in fusion events in MELTVR or other INI1/*SMARCB1*-deficient tumors (e.g., epithelioid sarcoma, malignant rhabdoid tumor). This fusion involves a breakpoint in exon 4 of *GSTT1* (transcript NM_000853.4) at chr22:24376861 (GRCh37/hg19), and the 3’ partner *IGLC7* (RefSeq NG_000002) with a breakpoint at chr22:23273234 (GRCh37/hg19; no canonical transcript annotated). Structurally, this fusion disrupts the functional C-terminal domain of *GSTT1*, whereas *IGLC7* lacks any recognized oncogenic domains. Thus, the biological significance of the *GSTT1::IGLC7* fusion is uncertain at this time and may represent a passenger genetic alteration.

MELTVR must be differentiated from several histologically and immunophenotypically similar tumors. Soft tissue myoepithelial tumors are most frequently located in the subcutaneous tissues of acral regions and proximal limb girdles and may extend into the dermis. Their histological features overlap with those of MELTVR, being composed of spindle, oval, or epithelioid cells arranged in solid, reticular, or cord-like patterns within a myxoid stroma. A subset of cases also exhibits INI1/*SMARCB1* deficiency (12). However, these tumors typically co-express epithelial markers and S-100. Most harbor *EWSR1* gene rearrangements, with a minority containing *FUS* rearrangements (13). Epithelioid sarcoma, particularly the proximal type, may also involve the vulva. The tumor cells are epithelioid with vesicular nuclei and show loss of INI1/*SMARCB1* expression. Proximal-type epithelioid sarcoma exhibits infiltrative growth, marked nuclear atypia, and frequent necrosis, while myxoid change is uncommon. Tumor cells generally display diffuse CK and CD34 expression and lack ER expression (14), allowing distinction from MELTVR. Malignant rhabdoid tumor can arise in the vulva and also shows loss of *SMARCB1*/INI1 protein expression. However, these tumors primarily occur in infants and young children and are exceedingly rare in adults. They typically progress rapidly, presenting as large, unencapsulated masses. Histologically, the tumor cells exhibit rhabdoid morphology, with eosinophilic cytoplasm, prominent nucleoli, and eccentric nuclei, and are frequently associated with necrosis and numerous mitotic figures. These tumors express CK and EMA and show a high Ki-67 proliferation index (3). Extraskeletal myxoid chondrosarcoma is another entity that rarely arises in the vulva. Similar to MELTVR, it features a myxoid stroma, and previous studies have reported that some cases exhibit loss of INI1/*SMARCB1* expression (12). Nevertheless, its tumor cells predominantly express Vimentin but lack EMA or ER expression. More than 90% of cases harbor *NR4A3* fusion genes (15), which are not detected in MELTVR. Aggressive angiomyxoma predominantly arises in the vulvar region of women and commonly expresses ER and PR. However, it typically presents as a large, deeply invasive, and unencapsulated lesion characterized by desmin-positive spindle cells within a myxoid stroma, and may harbor *HMGA2* gene rearrangements (16). For a clear comparison between MELTVR and its major differential diagnoses, the core features are summarized in **Table 1**.

**Table 1:** Comparison of key features between MELTVR and its major differential diagnoses.

Tumor type	Histologic features	Immunophenotype	Molecular genetics
MELTVR	Lobular architecture; epithelioid/spindle cells; myxoid and non-myxoid stroma	Loss of INI1 expression; Vimentin, EMA, ER positive; CD34, Desmin and CK negative	*SMARCB1* deletion
Soft tissue myoepithelial tumor	Spindle or epithelioid cells; myxoid stroma	Co-expression of epithelial markers and S-100; ER negative	*EWSR1*/*FUS* rearrangement
Proximal-type epithelioid sarcoma	Infiltrative growth; marked nuclear atypia; frequent necrosis; uncommon myxoid change	Loss of INI1; CK and CD34 positive; ER negative	*SMARCB1* deletion
Malignant rhabdoid tumor	Rhabdoid cell morphology; brisk mitoses and necrosis	Loss of INI1; CK and EMA positive; high Ki-67 index	*SMARCB1* deletion
Extraskeletal myxoid chondrosarcoma	Prominent myxoid stroma; relatively monomorphic cells	Loss of INI1 in subset; Vimentin positive; EMA, ER negative	*NR4A3* rearrangement
Aggressive angiomyxoma	Large, deeply invasive, spindle cells in myxoid stroma	ER, PR and desmin positive	*HMGA2* rearrangement

References are marked in the original text and not repeated here.

In current clinical practice, the primary treatment for MELTVR is complete surgical resection; adjuvant radiotherapy or chemotherapy is generally deemed unnecessary postoperatively (17). Literature reports indicate that two patients treated with only intralesional excision or enucleation experienced local recurrence, highlighting the importance of negative surgical margins for local tumor control. Although three patients developed tumor regrowth or late recurrence at 12 years postoperatively, none exhibited destructive growth, and all remained alive without distant metastasis at a mean follow-up of 66 months (1). Even in the case of an extremely large 12-cm lesion, no postoperative complications were observed 36 months after extensive resection following two recurrences (18). Collectively, these follow-up data indicate that MELTVR generally exhibits relatively indolent biological behavior. Despite its potential for local invasiveness and recurrence, the overall prognosis is favorable.

Nonetheless, several uncertainties persist in the prognostic evaluation of MELTVR. For instance, one patient with extensive tumor infiltration into adipose tissue and positive surgical margins exhibited neither recurrence nor metastasis during 12 months of postoperative follow-up (5). Moreover, some MELTVR cases with malignant-like pathological features (e.g., high-grade nuclear atypia, increased mitotic activity, vascular invasion) showed no evidence of metastasis during follow-up (1, 6). These observations suggest that such factors may not be reliable indicators for assessing the malignant potential of MELTVR. Given the limited number of reported cases and the relatively short follow-up duration in certain studies, potential biases may influence the assessment of long-term prognosis. The independent prognostic value of these pathological characteristics, previously hypothesized to predict poor outcomes, can only be elucidated through the accumulation of additional cases and long-term follow-up investigations.

## Conclusion

As a rare *SMARCB1*/INI1-deficient vulvar mesenchymal tumor, MELTVR is molecularly characterized by *SMARCB1* gene deletion and the absence of fusion genes such as *EWSR1*, *FUS*, and *NR4A3*. This study makes two novel contributions. First, we report a rare case of ER-negative MELTVR, challenging the long-standing notion that ER expression is consistently positive in this tumor type. Second, we identified the *GSTT1::IGLC7* gene fusion for the first time, expanding the known molecular genetic spectrum of MELTVR. From a clinical oncology perspective, these findings broaden the recognized immunophenotypic and molecular diversity of MELTVR. Awareness of this variability is important for accurate diagnosis. For future research, larger cohort studies are warranted to determine the incidence of the ER-negative phenotype and *GSTT1::IGLC7* fusion in MELTVR, as well as their potential clinical and prognostic significance.

## Data Availability

The original contributions presented in the study are included in the article/supplementary material. Further inquiries can be directed to the corresponding author.
